# A systematic review on functional electrical stimulation based rehabilitation systems for upper limb post-stroke recovery

**DOI:** 10.3389/fneur.2023.1272992

**Published:** 2023-12-08

**Authors:** Muhammad Ahmed Khan, Hoda Fares, Hemant Ghayvat, Iris Charlotte Brunner, Sadasivan Puthusserypady, Babak Razavi, Maarten Lansberg, Ada Poon, Kimford Jay Meador

**Affiliations:** ^1^Department of Neurology and Neurological Sciences, Stanford University, Palo Alto, CA, United States; ^2^Department of Electrical Engineering, Stanford University, Palo Alto, CA, United States; ^3^Department of Health Technology, Technical University of Denmark, Lyngby, Denmark; ^4^Department of Electrical, Electronic, Telecommunication Engineering and Naval Architecture (DITEN), University of Genoa, Genoa, Italy; ^5^Department of Computer Science, Linnaeus University, Växjö, Sweden; ^6^Department of Clinical Medicine, Hammel Neurocenter, Aarhus University, Hammel, Denmark

**Keywords:** stroke, rehabilitation, functional electrical stimulation (FES), upper limb neurorehabilitation, post-stroke therapy, stroke rehabilitation systems

## Abstract

**Background:**

Stroke is one of the most common neurological conditions that often leads to upper limb motor impairments, significantly affecting individuals' quality of life. Rehabilitation strategies are crucial in facilitating post-stroke recovery and improving functional independence. Functional Electrical Stimulation (FES) systems have emerged as promising upper limb rehabilitation tools, offering innovative neuromuscular reeducation approaches.

**Objective:**

The main objective of this paper is to provide a comprehensive systematic review of the start-of-the-art functional electrical stimulation (FES) systems for upper limb neurorehabilitation in post-stroke therapy. More specifically, this paper aims to review different types of FES systems, their feasibility testing, or randomized control trials (RCT) studies.

**Methods:**

The FES systems classification is based on the involvement of patient feedback within the FES control, which mainly includes “Open-Loop FES Systems” (manually controlled) and “Closed-Loop FES Systems” (brain-computer interface-BCI and electromyography-EMG controlled). Thus, valuable insights are presented into the technological advantages and effectiveness of Manual FES, EEG-FES, and EMG-FES systems.

**Results and discussion:**

The review analyzed 25 studies and found that the use of FES-based rehabilitation systems resulted in favorable outcomes for the stroke recovery of upper limb functional movements, as measured by the FMA (Fugl-Meyer Assessment) (Manually controlled FES: mean difference = 5.6, 95% CI (3.77, 7.5), *P* < 0.001; BCI-controlled FES: mean difference = 5.37, 95% CI (4.2, 6.6), *P* < 0.001; EMG-controlled FES: mean difference = 14.14, 95% CI (11.72, 16.6), *P* < 0.001) and ARAT (Action Research Arm Test) (EMG-controlled FES: mean difference = 11.9, 95% CI (8.8, 14.9), *P* < 0.001) scores. Furthermore, the shortcomings, clinical considerations, comparison to non-FES systems, design improvements, and possible future implications are also discussed for improving stroke rehabilitation systems and advancing post-stroke recovery. Thus, summarizing the existing literature, this review paper can help researchers identify areas for further investigation. This can lead to formulating research questions and developing new studies aimed at improving FES systems and their outcomes in upper limb rehabilitation.

## 1 Introduction

Stroke occurs when blood flow to the brain is acutely compromised, resulting in neural injuries and subsequently functional impairment and sometimes long-term disabilities (1, 2). This life-changing event can significantly impair cognitive, emotional, and physical functions. Studies show that individuals convalescing from a stroke frequently experience feelings of frustration, helplessness, and social isolation, which can lead to a higher risk of depression and a reduced capability to perform daily activities (3, 4). A study estimated that in 2016, stroke caused approximately 5.5 million deaths and 116.4 million DALYs (disability-adjusted life-years) worldwide (5). Among stroke survivors, upper limb hemiparesis, i.e., weakness or lack of ability to move the upper limb on one side of the body is a common condition (6). Further, ~55–75% of stroke patients with a hemiplegic arm still have a defective function in arm movements after 3 to 6 months of rehabilitation (7).

Post-stroke care primarily aims to rehabilitate patients to effectively recover lost functions and help them in their daily activities. This allows them to have their independence and reintegrate into society. Among different rehabilitation methods, occupational and physical therapies are the most common stroke rehabilitation methods for restoring motor functions (8). These approaches use task-specific and repetitive training to induce motor recovery, leveraging innate motor learning and neuroplasticity mechanisms. However, functional recovery is not always satisfactory, as only 20% of patients are fully able to resume their social life after physical rehabilitation (9). This shows a significant gap in the overall effectiveness of the rehabilitation and recovery processes, thus indicating the need for new approaches to restore patients' functional mobility and ultimately improve their quality of life (10).

**Figure 1:**
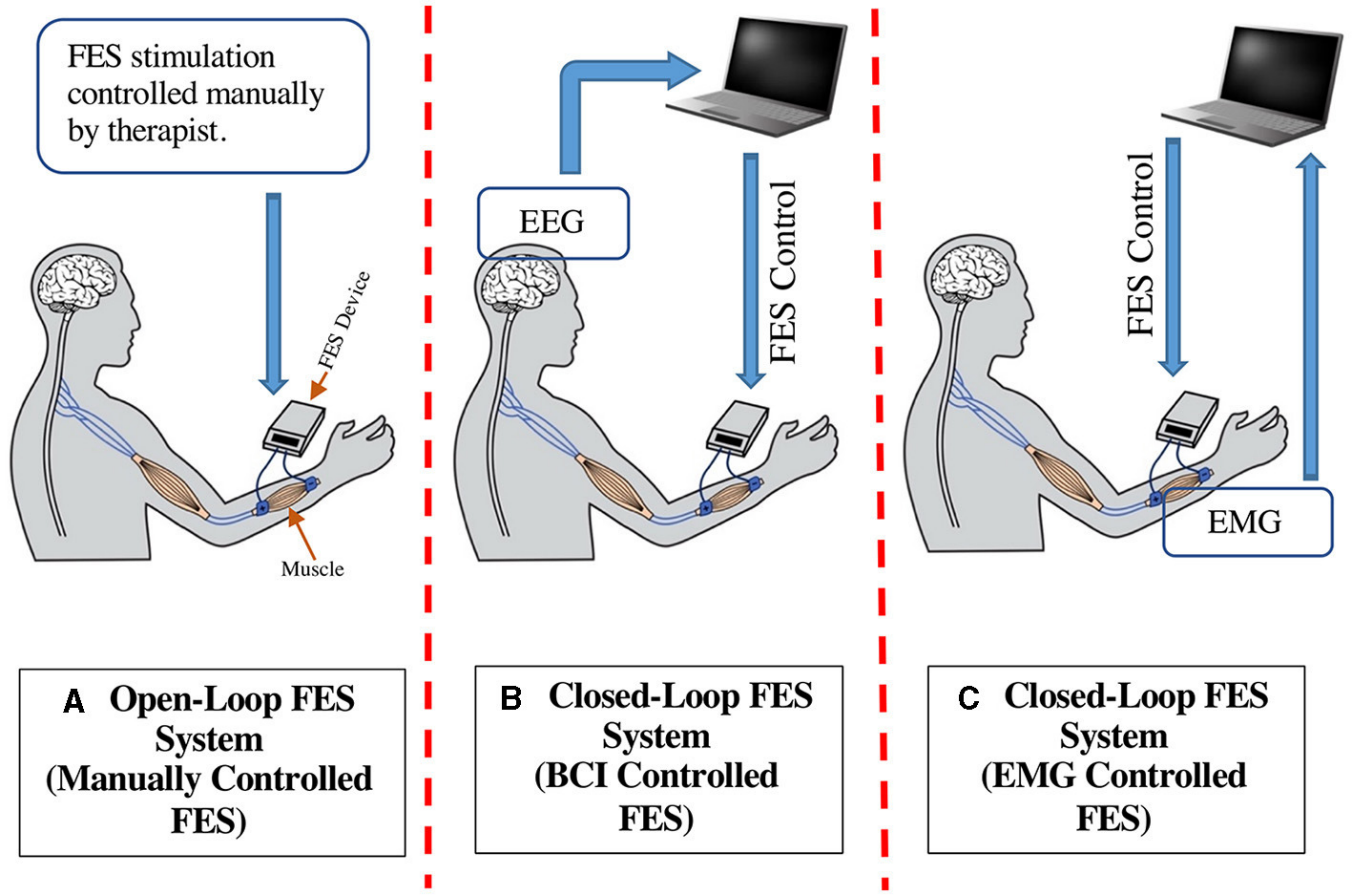
Types of FES based rehabilitation system for stroke recovery **(A)** Open-Loop FES System, **(B)** Closed-Loop FES System (BCI-FES), **(C)** Closed-Loop FES System (EMG-FES).

Advancements in science and technology have introduced different stroke rehabilitation methods, among which the functional electrical stimulation (FES) is commonly being used (11). FES is a rehabilitation tool for restoring the motor skills of stroke survivors by applying electrical impulses through the skin surface to stimulate targeted nerves, thus instigating movements in paretic muscles (12–14). Electrical stimulation applied to the muscle is controlled so that the movement produced will provide a useful function and not a random trajectory. Depending on their mode of operation, FES systems fall into 2 major types: Open-loop FES and Closed-loop FES systems (Figure 1). In open-loop systems, FES is mainly applied by a therapist using preprogrammed patterns that cannot be controlled by the patient feedback to initiate the muscle activation (Figure 1A). Open-loop FES was first introduced to hemiplegia patients by Moe and Post (15) and later improved by Kralj et al. to treat patients with neural disorders (16). Many studies have validated the efficacy of open-loop FES in upper limb stroke rehabilitation application (17–24).

Rehabilitation therapies aim to restore brain connections that subserve motor recovery and function. Along with the therapist's assistance, the patient's active participation via feedback loop can further improve recovery outcomes. In this regard, closed-loop FES systems play a substantial part, mainly including brain-computer interface (BCI) and electromyogram (EMG) controlled FES systems (Figures 1B, C, respectively). In BCI-FES (also called electroencephalogram (EEG)-FES), motor imagery (MI) paradigms facilitate an effective approach to neurorehabilitation (25–28). A BCI system provides a direct interaction channel between the brain and a peripheral device by translating the brain's electrical activities (as captured by EEG) into control/command signals. For rehabilitation application, the MI training consists of representing imaginary movements of limbs without physically performing them. During rehabilitation, the MI activates the neural circuits involved in actual movements and could induce functional redistribution of neuronal circuits through neural plasticity (29–31). An MI-BCI is a computer-based system that records the EEG signals and translates the user's intention to perform the specific task based on MI events. Thus, the EEG signal is used to generate a muscle electrical stimulation pattern that matches the intended movements of user (the user imagines and tries to perform that movement). Such MI-BCI methods with FES systems have widely been used in stroke rehabilitation for motor and functional recovery (32–45).

Besides EEG, EMG-controlled FES has been proven to be an efficient method for stroke rehabilitation. EMG signal measures the electrical currents generated in muscles during their contraction, representing neuromuscular activity (46). Using EMG as feedback in the EMG-FES device enables real-time analysis of muscle activity and adjusts the amount of FES stimulation based on the muscle's requirement (47–49). Thus, the resulting movement and intrinsic multisensory activation are paired with the subject's active attention and intention. Furthermore, the muscle contraction is modulated by the subjects themselves, hence, facilitating fast motor learning and recovery of lost function. Finally, EMG-controlled FES limits the chances of excess electrical stimulation of muscles, which otherwise can cause muscle cramps and fatigue (50). Different studies have been performed to develop and test EMG-controlled FES systems for stroke rehabilitation applications (51–58).

To date, different review papers have been published regarding stroke rehabilitation, which include FES in rehabilitation engineering (59), the usability of FES in upper limb stroke rehabilitation (60), the effectiveness of upper limb FES after stroke (12), devices used in muscular electrical stimulation for stroke rehabilitation (61), EMG-triggered/controlled electrical stimulation for motor recovery of the upper limb (48), BCI systems for post-stroke rehabilitation (11, 62, 63), flexible technology in stroke rehabilitation systems (64), home-based technologies for stroke rehabilitation (65), efficacy of robotic exoskeleton for gait rehabilitation (66), game-based virtual reality system for upper limb rehabilitation (67), and different techniques to stimulate upper extremity stroke recovery (68). However, no review article lists and discusses the different types of FES systems for upper limb stroke rehabilitation. Hence, in this systematic review, we assessed the RCT, and feasibility testing studies related to different FES-based rehabilitation systems to determine their impact on improving upper limb functional movements among stroke patients. By examining the effectiveness and implications of various FES approaches, this review also provides a comprehensive overview of the potential benefits and challenges associated with FES-based stroke rehabilitation, offering insights into the future direction of this promising therapeutic modality.

## 2 Methods

We followed the PRISMA (Preferred Reporting Items for Systematic Reviews and Meta-Analyses) guidelines for the systematic review. Three researchers independently performed the search strategy, eligibility criteria, and data extraction of included studies.

### 2.1 Search strategy

**Figure 2:**
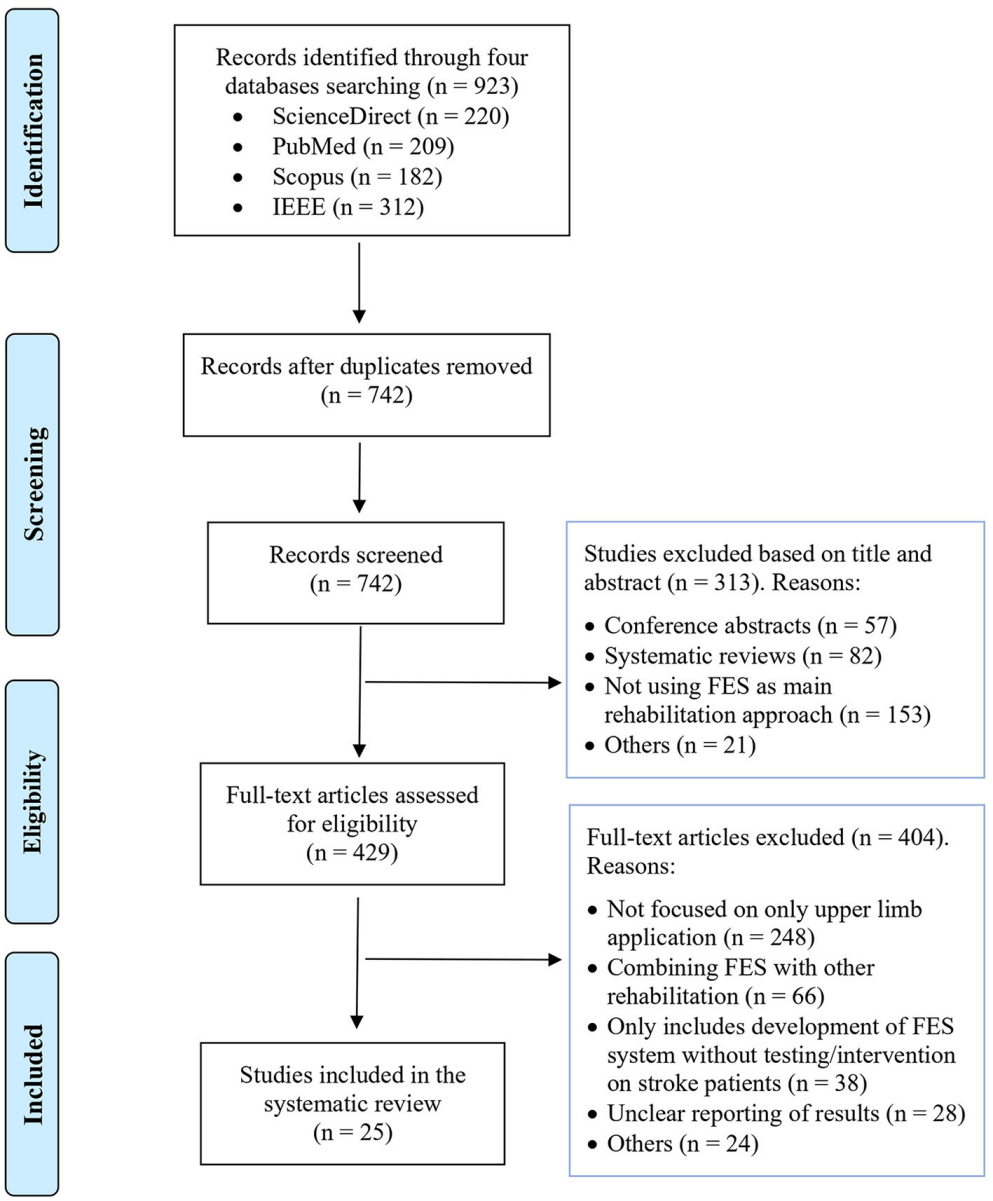
PRISMA flow chart of study selection.

The review was conducted using four academic electronic databases including ScienceDirect, PubMed, Scopus, and IEEE databases using the keywords: stroke rehabilitation, functional electrical stimulation (FES), RCT, feasibility testing, upper limb functional movements, brain-computer interface (BCI), EMG-based rehabilitation, BCI-based rehabilitation, EEG-based rehabilitation, neurorehabilitation devices, upper limb rehabilitation, EMG-controlled FES, BCI-controlled FES, EEG-controlled FES. Figure 2 illustrates the PRISMA flow chart of study selection. Initially, 923 research articles were found from a keyword search in the different databases. Among them, 181 duplicates were removed. Then, the remaining 742 papers were evaluated, and based on their titles and abstract, 313 articles were excluded. Lastly, full-text screening was performed and only 25 manuscripts fulfilled the inclusion criteria and were included in this review paper. Of these, 8, 13, and 4 manuscripts involved open-loop FES, closed-loop BCI/EEG-FES, and closed-loop EMG-FES systems, respectively.

### 2.2 Eligibility criteria

A systematic search was performed based on predefined inclusion criteria (IC) and exclusion criteria (EC). In the final stage, only those research papers were selected that met all the conditions listed below:

IC1: Written in English.
IC2: Published on or after the year 2009.
IC3: Related to FES-based stroke rehabilitation in terms of “Manually operated” OR “BCI/EEG controlled” OR “EMG controlled”.
IC4: Focus on upper limb stroke rehabilitation.
IC5: Have validated the system performance on stroke patients (feasibility study OR RCTs).
EC1: Application other than stroke.
EC2: For lower limb stroke rehabilitation.
EC3: Testing only on healthy individuals.

### 2.3 Data extraction

Three authors independently extracted the following information of each included study: type of used rehabilitation system, experimental and control groups, application as RCT/feasibility study, upper limb targeted areas, total number of therapy sessions, therapy session time and outcome measures/performance evaluation. Any disagreement during the process of data extraction were resolved through discussion among three authors.

### 2.4 Quality assessment

The methodological quality and risk of bias of the included studies were assessed using validated tools. For randomized controlled trials, the Risk of Bias 2 (ROB2) tool was used to evaluate potential bias across five domains–randomization, deviations from intervention, missing outcome data, outcome measurement, and selection of reported results (69). The quality of observational case series studies was appraised using the NIH Quality Assessment tool, which contains 9 items assessing aspects like study objective, population description, intervention clarity, outcome validity, and follow-up (70). Finally, the included case reports were critically appraised using the Joanna Briggs Institute (JBI) checklist for case reports (71). This tool evaluates key domains such as patient demographics, clinical history, diagnosis assessment, intervention details, and outcome measures.

### 2.5 Statistical analysis

Continuous data was analyzed using OpenMetaAnalyst software. A fixed effect model calculated mean differences (MD) with 95% confidence intervals (CI) for continuous outcomes. Statistical homogeneity and heterogeneity were assessed using the I^2^ statistic. An I^2^ value >50% was considered indicative of substantial heterogeneity.

## 3 Results

### 3.1 Risk of bias in included studies

Among a total of 25 included studies, the quality of the 9 RCTs (17, 32–36, 51, 55, 56) was assessed using the “Risk of Bias 2 (ROB2)” tool (Supplementary Figures S1, S2). Overall, most studies were rated as having a low risk of bias in terms of the randomization process, missing outcome data, and measurement of the outcome. However, in 4 studies (17, 32, 33, 35), some concerns were identified regarding deviations from intended interventions, resulting in a rating of “some concerns.” The remaining five studies (34, 36, 51, 55, 56) were found to have an overall “low risk” of bias.

The quality of the 11 observational case series studies (18–22, 24, 41, 43–45, 58) was assessed using the “NIH Quality Assessment” tool. Based on the assessment, 7 studies were determined to be of “good quality”, while 4 studies were evaluated as “fair quality” (Supplementary Table S1). In addition, five case reports (23, 37, 38, 40, 42) were included and appraised using the “Joanna Briggs Institute (JBI) Critical Appraisal” tool. The overall quality of each case report was “good” based on the JBI checklist (Supplementary Table S2).

### 3.2 Included studies regarding types of FES-based stroke rehabilitation systems

#### 3.2.1 Open-loop FES system: pre-defined FES for stroke rehabilitation

An open-loop FES system comprises of a manually controlled device, which is operated by a therapist. During the therapy session, the therapist manually administers electrical stimulation to the specific muscles of the patient, using patient-specific predetermined stimulation parameters such as stimulation intensity, time duration, and ON/OFF cycle.

Numerous studies have been conducted utilizing open-loop FES systems for stroke rehabilitation to regain upper limb motor functions. Experimenting with the effectiveness of FES, Nakipoglu Yuzer et al. (17) applied FES (two channels and four surface electrodes) to the spastic muscles of 30 patients (an RCT), and the improvement of clinical scores indicates that FES effectively reduces wrist flexor spasticity. In (18), Makowski et al. showed that FES produces functional hand opening when the patient is relaxed, but it is overpowered by finger flexor coactivation when the patient voluntarily exerts effort to reach/open the hand. For that, their study proves that the amount of hand opening grows significantly (3.2–8.8 cm) when including FES for both reaching and hand opening muscles even in the presence of submaximal or zero effort. Moreover, Meadmore et al. (19) investigated FES of shoulder, elbow, and wrist muscles: five patients underwent 18 sessions and completed FMA (Fugl-Meyer Assessment) and ARAT (Action Research Arm Test) assessments. The study showed an improvement of 4.4, providing evidence that the integration of low-cost hardware with advanced FES controllers can reduce upper limb impairment. Sun et al. (20) reported the FES for upper limb functional activity practice, used by 9 therapists to set 8 sessions activities with 22 stroke patients. Among them, 17 patients showed a session completion rate >90%, demonstrating its capability of delivering high-intensity therapy compared to traditional face-to-face therapy. Also, Niu et al. (21) illustrated a technique for creating FES patterns based on muscle synergies of a normal subject (three patients–adjusted for each participant and task) using a programmable FES device. Followed by 5-day sessions of intervention using synergy-based FES delivery to another three patients. The outcome of the new technology was measured by improvement in FMA scores (28.6% ± 13.7%). In Chou et al. (22), made use of the latter in the design and test of an automated synergy-based FES system to match electrically induced movements to assist residual movements of patients. Results based on changes in FMA scores indicate that the synchronization produced more consistent compound movements with reduced RMS (root mean square) errors under different triggering conditions. Martín-Odriozola et al. (23) developed the Fesia Grasp device used for hand dexterity rehabilitation of a 69-yearold post-ischemic stroke woman. Following their first study (21), Niu et al. (24) conducted a TOT (Task-oriented training) protocol with repeated forward and lateral reaching movements assisted by synergy-based FES on 16 patients, divided into FES (EG) and Sham (CG) groups over 5-days. Findings of higher FMA than Sham indicate efficacy of open-loop FES system in post-stroke rehabilitation. A detailed overview of research studies regarding open-loop FES rehabilitation is provided in Table 1.

**Table 1:** Research studies and their outcomes for open-loop FES neurorehabilitation systems.

**Open-loop FES systems for upper limb stroke rehabilitation**				
**Study**	**Commercial/ customized open-loop FES rehabilitation system**	**Experimental group (EG) and control group (CG)**	**i. Upper limb targeted areas ii. Total sessions iii. Therapy time/session**	**Outcome measures/ performance evaluation/other comments**
Nakipoglu Yuzer et al. (17)	Customized	RCT EG and CG: 30 post-stroke hemiplegic patients were randomly divided into EG and CG. FES was only applied to EG.	i. Wrist and finger extensors for wrist flexor spasticity ii. 20 iii. 30 min	ΔBI (EG) = 6.34 ± 1.06 ΔBI (CG) = 3 ± 1.02 ΔRMA (EG) = 0.66 ± 0.2 ΔRMA (CG) = 0.34 ± 0.31 ΔUEFT (EG) = 0.4 ± 0.28 ΔUEFT (CG) = 0.2 ± 0.08 ΔAROM (EG) = 6.73 ± 0.56 ΔAROM (CG) = 2.47 ± 0.62 ΔMAS3 (EG) = 80%-46.7% = 33.3% ΔMAS3 (CG) = 46.7%-40% = 6.7% A significant difference was found in favor of EG
Makowski et al. (18)	Customized	Feasibility study EG: 5 at least 6-months post-stroke patients.	i. Reaching and hand opening muscles ii. At least 3 sessions per patient iii. N/A	Hand opening average of participants increased significantly when including FES for reaching and hand opening in the presence of partial or zero reaching effort: (“+” sign shows the combination of two states) HE + RE = 3 cm (no stimulation) HE + RES = 3.2 cm RES + HES = 6.5 cm RES + HS = 8 cm RS + HS (0 effort - relaxed) = 8.8 cm
Meadmore et al. (19)	Customized (feasibility study)	Feasibility study EG: 5 stroke patients with hemiplegia CG: the same 5 patients completed 5 un-assisted tasks.	i. Shoulder, elbow and wrist muscle groups. ii. 18 sessions iii. 1 h	The FMA and ARAT were completed 1-6 days pre and post-intervention. Improvement was significant for both tests (Mean Results): ΔFMA (EG) = 23.2-18.8 = 4.4 ΔARAT (EG) = 7-2.6 = 4.4
Sun et al. (20)	Commercial FES-UPP flexible system (5 channels—FSM controller—feedback software)	Feasibility study EG: 22 patients with impaired upper limbs	i. Upper limb muscles ii. 8 tailored sessions per participant iii. N/A	Mean efficiency and mean number of successful repetitions of activities (NSR) in: Session 1: 12% Efficiency and 13 NSR Session 7: 34% Efficiency and 45 NSR 17 of 22 participants had a therapy completion rate >90%
Niu et al. (21)	Customized (Synergy based FES Device)	Feasibility study EG: 6 (3 pattern adjustment and 3 synergy-based FES testing)	i. Upper limb muscles ii. 5 iii. 1 h	Mean value of the change in FMA scores pre and post treatment indicate the improvement in functional movement. ΔFMA = 5.7 ± 2.5 (28.6% ± 13.7% change)
Chou et al. (22)	Customized (Automated FES System based on synergy FES)	Feasibility study EG: 5 patients (4 ischemic and 1 hemorrhagic all >MAS2) to test the system and 4 healthy patients to adjust the patterns	i. Upper limb muscles ii. 5 iii. 1 h	The lowest RMS errors of subjects (S0) under different trigger levels (TL) in each task (Forward or Lateral Reaching): S02 (TL 0.3) FR: 0.796 ± 0.290 S03 (TL 0.5) FR: 0.511 ± 0.190 S04 (TL 0.2) FR: 0.499 ± 0.227 S05 (TL 0.2) LR: 0.810 ± 0.372 S06 (TL 0.5) LR: 0.732 ± 0.213
Martín-Odriozola et al. (23)	Commercial (multi-field fesia grasp system FES)	Feasibility study EG: 69-year-old post ischemic stroke woman	i. Left hand dexterity. ii. 12 iii. 1 hour	ΔAROM (thumb) = +27° ΔAROM (index) = +8° ΔAROM (wrist) = +24° ΔGS = 2.9 kg
Niu et al. (24)	Customized	Feasibility study 16 patients with post-stroke hemiparesis EG: 9 FES CG: 7 Sham	i. 7 upper extremity muscles of elbow and shoulder ii. 5 iii. 1 h	FMA-UE scores of patients receiving FES increased by 6.67 ± 5.20 (28.13 ± 21.41%) FMA-UE scores of patients receiving Sham changed by 2.00 ± 2.38 (7.32 ± 16.11%)

HE, Max Hand Opening Effort; RE, Max Reaching Effort; RS, Reaching Stimulation; HS, Hand Opening Stimulation; RES, Partial Reaching Effort and Stimulation; HES, Partial Hand Opening Effort and Stimulation; FMA, Fugl-Meyer Assessment; ARAT, Action Research Arm Test; BI, Barthel Index; RMA, Rivermead Motor Assessment; UEFT, Upper Extremity Functional Index; AROM, Active Range of Motion; MAS, Motor Assessment Scale; NSR, Number of Successful Repetitions of Activities; RMS, Root Mean Square; FR, Forward Reaching; LR, Lateral Reaching; UE, Upper Extremity; GS, Grip Strength.

#### 3.2.2 Closed-loop FES system: BCI controlled FES for stroke rehabilitation

According to Hebb's principle “cells that fire together wire together” (72, 73), suggesting that the coordination of cortical and physical activities during the rehabilitation therapies could lead to an effective improvement of the impaired motor function (74–77). Therefore, a more effective approach may be to interface the FES rehabilitation device with an external system that could enhance the simultaneous activation of the motor cortex during the rehabilitation sessions. In this regard, MI-based BCI systems are an optimum choice, which allows the rehabilitation system to perform the required task based on the patient's imagination of intended motion, allowing more active participation of brain throughout the stroke therapy (78).

**Figure 3:**
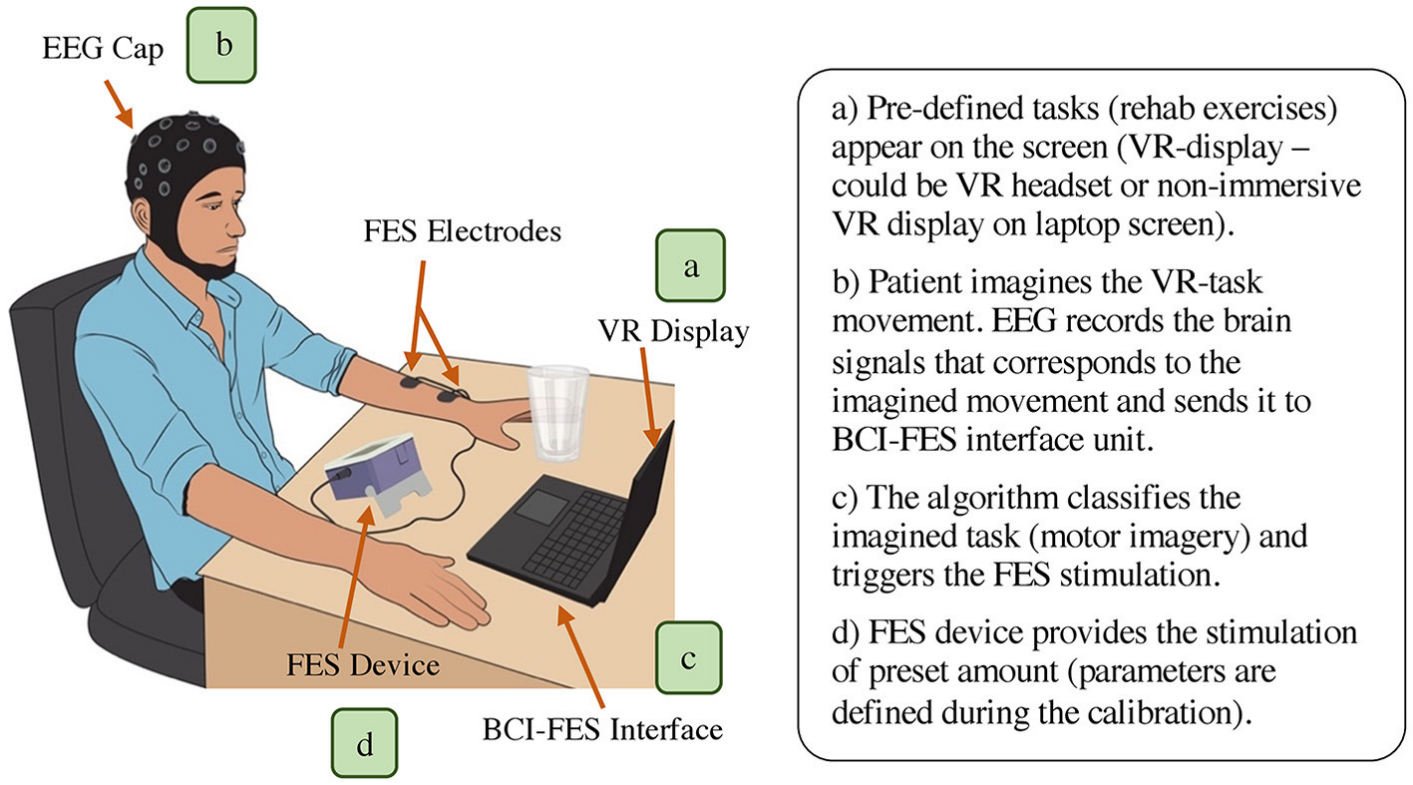
Overall representation of BCI-FES neurorehabilitation system (main components: EEG unit for BCI, BCI-FES interface, and FES module. Optional component: VR display/headset).

A BCI-FES rehabilitation system mainly comprises of a BCI unit (containing EEG element), BCI-FES interface component, and FES module. Some BCI-FES systems also incorporate a virtual reality (VR) paradigm as a part of their setup (Figure 3). In such systems, firstly, the patient is provided the VR environment (on screen or the headset) that contains the pre-programmed therapy session of targeted motion e. g., hands extension/flexion (Figure 3A). The patient will be asked to imagine the task execution displayed in the virtual environment. Each task imagination generates a specific EEG signal, which will be acquired and processed by the BCI unit (Figure 3B). Depending on the imagination, the BCI-FES interface generates a trigger command to control the ON/OFF state of FES stimulation and will also control the stimulation parameters (Figure 3C). Lastly, the FES device provides electrical stimulation to the impaired muscles and hence, facilitate performing the required movements (Figure 3D). To make the strategy effective, the user typically undergoes training to establish a connection between their brain signals and specific motor tasks. This training involves practicing mental tasks or visualizing movements to generate distinct brain patterns that the BCI can recognize and translate into commands for the FES device.

BCI-FES systems are widely used for stroke rehabilitation and several randomized controlled trial (RCT) studies have been performed to investigate the efficiency of BCI-FES systems (32–36). In (32), Cincotti et al. performed an (RCT) to restore hand grasping movements. To assess post-stroke motor recovery, the FMA, MRC (Medical Research Council), and ESS (European Stroke Scale) scores were used. The results showed that the group with BCI-FES therapy achieved better motor recovery than the conventional FES group. Likewise, Li et al. (33) targeted stroke survivors with severe upper extremity paralysis. The study compared the efficiency of the BCI-FES system in comparison to the conventional FES system. The result showed a motor imagery task classification accuracy of 77%, along with a substantial improvement in the rehabilitation outcome scores within the BCI-FES group. In (34), Kim et al. accomplished an RCT to investigate the positive influence of the BCI-FES system on the motor recovery of upper extremities in stroke survivors. The measured outcomes validated the enhanced recovery via a BCI-based system compared to physical training. Additionally, in Miao et al. and Chen et al. (35, 36), the clinical application of the BCI-FES stroke rehabilitation system has been proposed to promote and improve upper extremity movements, along with motor activity restoration.

In addition to RCT, different feasibility studies for exploring the applicability of BCI-FES systems have also been carried out (37–45). In Daly et al. (37), Daly et al. performed a pilot study in which they tested a customized BCI-FES system on a stroke survivor having a joint extension problem in the index finger. During the first rehabilitation session, results showed a higher classification accuracy of 97% and 83% for “attempted movement” and “imagined movements” respectively. With every session, the muscle movement was gradually improving and by the end of nine sessions, the finger extension motion was completely recovered. Additionally, Mukaino et al. (38) developed a BCI-controlled neuromuscular electrical stimulator and conducted a case study on a stroke survivor (finger movement) to examine the effectiveness of BCI in stroke therapy. The results indicated that rehabilitation training with a BCI-controlled FES induces cortical plasticity and promotes functional recovery. Apart from customized BCI-FES stroke rehabilitation systems, “RecoveriX from g.tec” is commercially available stroke rehab systems (39). The RecoveriX system classifies the right and left wrist motion intention and is only meant for the wrist dorsiflexion rehabilitation paradigm. Hence, to validate the efficacy of RecoveriX system, Sabathiel et al. (40), Irimia et al. (41), Cho et al. (42), Qiu et al. (43), and Sebastián-Romagosa et al. (44) have conducted experiments on a set of stroke survivors for arm function restoration. Their results showed that the system depicts a classification accuracy of up to 95%. Furthermore, significant improvements of upper limb motor function scores suggest the post-stroke motor recovery. A detailed overview of research studies regarding BCI-FES rehabilitation is provided in Table 2.

**Table 2:** Research studies and their outcomes for BCI-FES neurorehabilitation systems.

**BCI controlled FES systems for upper limb stroke rehabilitation (closed-loop system)**						
**Study**	**Commercial/ customized BCI-FES rehabilitation system**	**EEG device channels configuration**	**Experimental group (EG) and control Group (CG)**	**Therapy per participant (i. Total sessions, ii. Runs/session, iii. Trials/run or Trials/session)**	**i. Upper limb targeted areas ii. Therapy time/ session**	**Outcome measures/ performance evaluation/ other comments**
Cincotti et al. (32)	Customized	32 channels	RCT: EG: 08 stroke patients CG (with conventional FES therapy): 08 stroke patients	i. 12 ii. 4 iii. 20 (per run)	i. Hand grasping movement (FES to paralyzed hand) ii. N/A	FMA, MRC and ESS score show a good recovery of hand function with BCI system as compared to the control group. Exact values of these scores have not been reported
Li et al. (33)	Customized	16 channels (G.tec Guger Technologies, Graz, Austria)	RCT: EG: 08 stroke patients CG (with conventional FES therapy): 07 stroke patients (Stroke Severity: subacute of severe level)	i. 24 ii. N/A iii. 20 (per session)	i. Upper extremity movements (FES stimulated the affected hand) ii. 1–1.5 h	FMA and ARAT score shows significant motor improvement. ΔFMA (EG) = 12.7, ΔFMA (CG) = 6.7, ΔARAT (EG) = 18.0; ΔARAT (CG) = 7.6
Kim et al. (34)	Customized	16 channels (PolyG-I by Laxtha Inc., Daejeon, Korea)	RCT: EG: 15 stroke patients CG (with conventional physical therapy): 15 stroke patients (Stroke Severity: Chronic of moderate level)	i. 20 ii. N/A iii. N/A	i. Shoulder and wrist movement (FES stimulated the affected hand) ii. 30 minutes	Improvement in FMA, MAL, MBI, and ROM was found. ΔFMA (EG) = 7.9, ΔFMA (CG) = 2.9
Miao et al. (35)	Commercial RecoveriX (g.tec GmbH, Austria)	16 channels (g.tec GmbH, Austria)	RCT: EG: 8 stroke patients CG (with conventional physical therapy): 8 stroke patients (Stroke Severity: Chronic of different levels)	i. 3 ii. 2 iii. 60 (per run)	i. Left or right wrist dorsiflexion (FES applied to both hands) ii. N/A	Average imagined task classification accuracy of 72.9%. Improvement in FMA score was found. ΔFMA (EG) = 3.5; ΔFMA (CG) = 0.9
Chen et al. (36)	Customized	32 channels (Neuroscan, USA)	RCT: EG: 16 stroke patients CG (with neuromuscular stimulation): 16 stroke patients (Stroke Severity: Chronic phase)	i. 11 ii. As much as possible (depending on each patient) iii. 10 (per run)	i. Left or right wrist extension ii. 40 minutes	FMA and Kendall MMT scores of the BCI-FES group was significantly higher than that in the control group.
Daly et al. (37)	Customized	58 channels (SynAmps, Compumedics, El Paso, TX)	Feasibility study EG: 01 stroke patient CG: N/A (Stroke Severity: 10 months post-stroke: Chronic of moderate to severe level)	i. 9 ii. N/A iii. 150 (per session)	i. Index finger joint extension (FES provided to isolated index finger extension) ii. 1.6 h	High accuracy in imagined movements (83%) and attempted movements (97%). Participants were able to execute 26 degrees of isolated index finger metacarpophalangeal joint extension
Mukaino et al. (38)	Customized	N/A	Feasibility study EG: 01 stroke patient CG (with conventional FES therapy): Same patient (Stroke Severity: Chronic of severe level)	(Total there are 4 phases) i. 10 (for each phase) ii. N/A iii. 600 (for each phase) (per session)	i. Finger movement (FES applied to the paralyzed finger) ii. 1 h	BCI-FES system efficacy reported via FMA and MAS score. ΔFMA (EG) = 3.5; ΔFMA (CG) = 0.5
Sabathiel et al. (40)	Commercial RecoveriX (g.tec GmbH, Austria) (39)	24 channels (g.Hiamp device by g.tec GmbH, Austria)	Feasibility study EG: 02 stroke patients CG: N/A (Stroke Severity: Chronic of severe level)	i. 24 (patient 1) and 10 (patient 2) ii. N/A iii. N/A	i. Wrist dorsiflexion (FES applied to both affected and unaffected hands) ii. N/A	Higher classification accuracy obtained. Moreover, Nine-Hole Peg Test (9-HPT) is performed only of patient 1 and result shows steady improvement over about three months
Irimia et al. (41)	Commercial RecoveriX (g.tec GmbH, Austria)	45 channels (g.tec GmbH, Austria)	Feasibility study EG: 03 stroke patients CG: N/A (Stroke Severity: Chronic of severe level)	i. 24 ii. 6 iii. 40 (per run)	i. 120 left and 120 right hand movements (FES applied to both affected and unaffected hands) ii. N/A	High accuracy in task execution achieved (95% in at least one session) and Nine-Hole Peg Test (9-HPT) shows improved motor function.
Cho et al. (42)	Commercial RecoveriX (g.tec GmbH, Austria)	16 channels (g.LADYbird by g.tec GmbH, Austria)	Feasibility study EG: 02 stroke patients CG: N/A (Stroke Severity: Chronic of severe level)	i. 25 ii. 4 iii. N/A	i. Left or right wrist dorsiflexion (FES applied to both hands) ii. 25 60-min	Improved performance observed via FMA score (pre and post BCI) Patient 1: ΔFMA = 21.0 Patient 2: ΔFMA = 11.0
Qiu et al. (43)	Commercial RecoveriX (g.tec GmbH, Austria)	16 channels (g.tec GmbH, Austria)	Feasibility study EG: 10 stroke patients CG: N/A (Stroke Severity: Chronic of different levels)	i. 12 ii. 2 iii. 30 (per run)	i. Left or right wrist dorsiflexion (FES applied to both hands) ii. N/A	System accuracy of more than 95%. FMA score shows enhanced motor function recovery among 5 patients (pre and post BCI)
Sebastián-Romagosa et al. (44)	Commercial RecoveriX (g.tec GmbH, Austria)	16 channels (g.tec GmbH, Austria)	Feasibility study EG: 51 stroke patients CG: N/A (Stroke Severity: 45 Chronic and 6 subacute phase)	i. 25 ii. 3 iii. 80 (per run)	i. Left or right wrist dorsiflexion (FES applied to both hands) ii. 1 h	Significant increase in the motor function of affected upper limb (ΔFMA = 4.68) Reduction of the spasticity in the wrist and fingers (ΔMAS-wrist = −0.72 ΔMAS-fingers = −0.63)
Choi et al. (45)	Customized	32 channels (G.tec Guger Technologies, Graz, Austria)	Feasibility study EG: 08 stroke patients CG: N/A (Stroke Severity: Chronic phase)	i. 5 ii. N/A iii. 24 (per session)	i. Different tasks from right/left hand (FES applied to the affected hand) ii. 1 h	Average imagined task classification accuracy of 71.25%.

FMA, Fugl-Meyer Assessment; MRC, Medical Research Council; ESS, European Stroke Scale; ARAT, Action Research Arm Test; MAS, Modified Ashworth Scale; MAL, Motor Activity Log; MBI, Modified Barthel Index; ROM, Range of Motion; MMT, Manual Muscle Testing.

#### 3.2.3 Closed-loop FES system: EMG controlled FES for stroke rehabilitation

EMG provides information on the neural activity of muscles and can detect physical movement intentions. A method has been previously studied to engage a user during FES therapy by triggering the stimulation when a specific level of muscle activity is detected (79–83). In the “EMG triggered FES system”, the EMG signal acts as a switch to trigger the delivery of FES stimulation at a predetermined level when the EMG magnitude reaches a certain threshold. However, this approach only uses the user's muscle activity to trigger FES and has not been conclusively proven advantageous over the open-loop FES method (79–83). Thus, another system, an “EMG controlled FES system” has been adopted that among with an FES trigger, also modulates the FES intensity in proportion to the real-time EMG signal (84).

**Figure 4:**
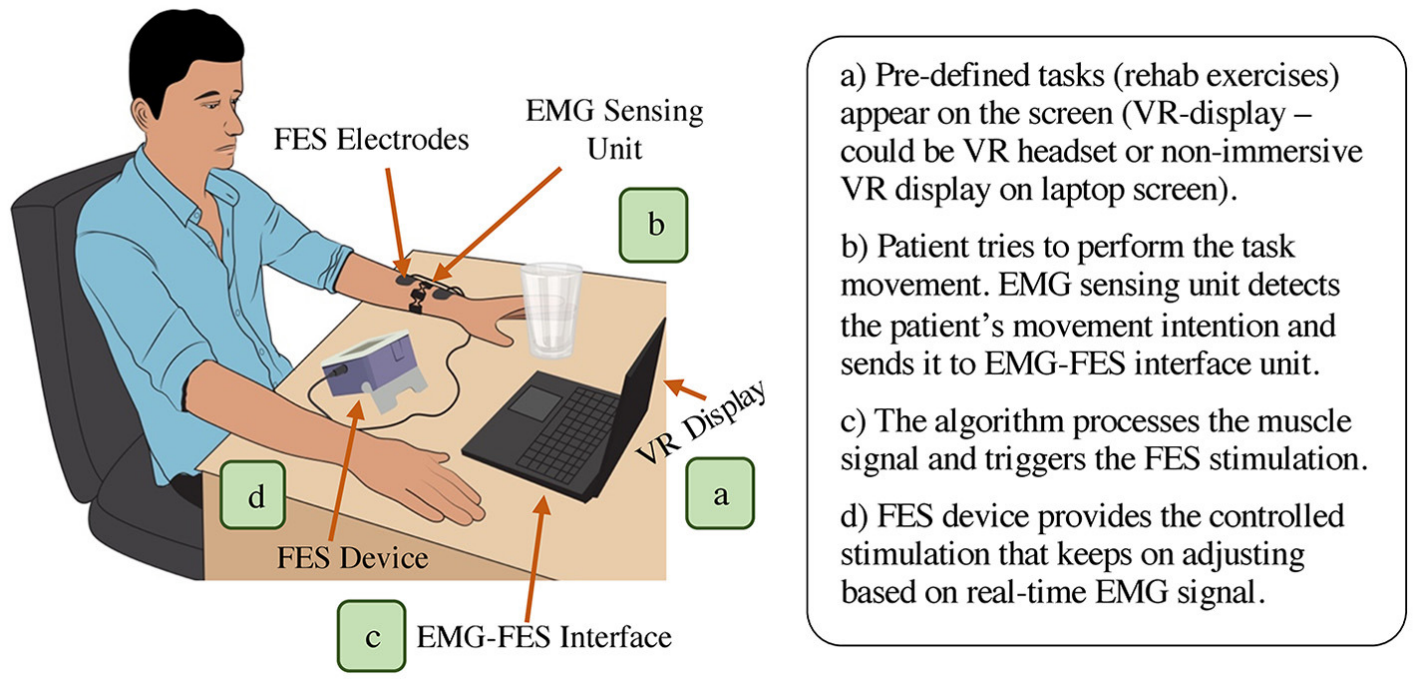
Overall representation of EMG-FES neurorehabilitation system (main components: EMG sensing unit, EMG-FES interface, and FES module. Optional component: VR display/headset).

EMG-controlled FES system mainly comprises of an EMG sensing unit, EMG-FES interface component, and FES module (Figure 4). Certain EMG-FES systems also integrate a virtual reality (VR) component into their configuration. In these systems, the initial phase entails immersing the patient in a VR environment, which can be presented on a screen or through a headset (Figure 4A). This VR environment includes a pre-programmed therapy session focused on specific movements, such as hand extension or flexion. Before the start of a therapy session, the system is calibrated for setting the EMG threshold level and required maximum FES stimulation (varies across subjects). The subject tries to perform the required task (for instance, wrist extension) and the intended motion is physically detected by the EMG sensing unit via analyzing the muscle activity (Figure 4B). The acquired EMG signal is processed by the EMG-FES interface and once the myoelectric activity reaches the pre-defined threshold level, the interface unit sends the trigger command to start the FES (Figure 4C). The applied stimulation activates the targeted muscle (or group of muscles) and helps the subject to achieve the desired motion (Figure 4D). In EMG-FES controlled system, the amount of stimulation does not stay constant and automatically adjusts throughout the therapy sessions proportional to real-time muscle activity.

Shindo et al. (51) performed an RCT to test the efficacy of the myoelectrical controlled electrical stimulator developed by Muraoka (52). The therapy sessions were performed for finger extension rehabilitation (i.e., a functional opening of the hand) which lasted for 3 weeks (5 days/week). The EMG electrodes were placed on the paretic extensor digitorum communis muscles and based on the muscle activities the amount of applied stimulation was controlled. After completion of rehabilitation sessions, pre, and post-performance was evaluated via different clinical score metrics (FMA and ARAT). They found that the EMG-controlled FES was able to induce a greater level of improvement as compared to the control group. In (53, 54), an EMG-controlled FES system, “MeCFES” has been developed by Thorsen's group for upper limb stroke rehabilitation, which was tested via RCT on 11 stroke survivors (55). In the experimental group, the EMG electrodes were placed on wrist and finger extensors and their recorded muscle activity was used to control the applied electrical stimulation for wrist and finger extension. The clinical evaluation was performed through the ARAT, and results showed that the participants treated with MeCFES had a significant improvement in upper limb motor function. In (56), Thorsen's group conducted another RCT in which they tested the MeCFES for task-oriented therapy (TOT). This was the first large RCT (68 stroke survivors) in which multiple rehabilitation centers validated the performance of MeCFES-assisted TOT against standard TOT. In the end, promising results were obtained in terms of MeCFES functioning, and no adverse events were reported in any of the centers. They concluded that MeCFES is a safe and efficient myo-controlled FES system for the motor recovery of upper extremities among stroke survivors. Recently (57), they developed an updated version of MecFES, named “FITFES”, which is wearable and portable in an ambulatory setting and best suitable for TOT applications. Thus far, only a working prototype has been tested on a single subject and no clinical evaluation has been performed. Moreover, Hara et al. (58) investigated the relationship between brain cortical perfusion (BCP) changes in the sensory-motor cortex (SMC) area and arm function improvement. A near-infrared spectroscopy (NIRS) approach was adopted to analyze BCP changes. It was found that EMG-FES rehabilitation improved FMA and GS (grip strength) scores. Also, NIRS showed increased SMC activation during therapy, confirming the functional improvement due to the EMG-FES system. A detailed overview of research studies using EMG-FES rehabilitation is provided in Table 3.

**Table 3:** Research studies and their outcomes for open-loop FES neurorehabilitation systems.

**EMG Controlled FES systems for upper limb stroke rehabilitation (closed-loop system)**				
**Study**	**Commercial/ customized EMG-FES rehabilitation system**	**Experimental group (EG) and control group (CG)**	**i. Upper limb targeted areas ii. Total sessions iii. Therapy time/session**	**Outcome measures/ performance evaluation/other comments**
Shindo et al. (51)	Customized (two channels EMG) (52)	RCT: EG: 12 stroke patients CG (physical and occupational therapy without FES): 12 stroke patients (Stroke Severity: stroke within 60 days of onset: Subacute level)	i. Fingers extension ii. 15 iii. N/A	Different clincal scores show significant motor improvement. ΔFMA (EG) = 12.2 ± 5.3; ΔFMA (CG) = 5.5 ± 6.0 ΔARAT (EG) = 13.2 ± 7.6; ΔARAT (CG) = 8.3 ± 8.1
Thorsen et al. (55)	Customized MeCFES (53, 54) (multi-channel EMG)	RCT: EG: 5 stroke patients CG (conventional FES without EMG): 6 stroke patients	i. Wrist and finger extension ii. 25 iii. 45 min	Improvement in ARAT score ΔARAT (EG) = 9.0; ΔARAT (CG) = 2.0
Jonsdottir et al. (56)	Customized MeCFES (53, 54) (multi-channel EMG)	RCT: EG: 32 stroke patients CG (task oriented standard therapy without FES): 36 stroke patients (Stroke Severity: Chronic and subacute level)	i. Task-oriented arm rehabilitation ii. 25 iii. 45 min	Improvement in clinical scores ΔFMA (EG) = 4.5; ΔFMA (CG) = 3.5 ΔARAT (EG) = 3.0; ΔARAT (CG) = 2.0
Hara et al. (58)	Commercial PAS System GD601 (t–wo channels EMG) (OG GIKEN Company, Okayama, Japan)	Feasibility study EG: 16 stroke patients CG: N/A (Stroke Severity: Chronic with moderate residual hemiparesis)	i. Supination and pronation, flexion and extension of individual fingers. Flexion and extension of the wrist. Flexion and extension of the elbow. Adduction and abduction of the shoulder. ii. 20-40 sessions iii. 40 min	Difference in pre and post rehabilitation scores show a good recovery of physical functions. ΔFMA = 20.0; ΔGS = 5.5 ± 11.0

FMA, Fugl-Meyer Assessment; ARAT, Action Research Arm Test; GS, Grip Strength.

### 3.3 Meta-analysis interpretation

#### 3.3.1 Change in fugl-meyer assessment (FMA) score

Among open-loop FES systems, the pooled analysis of 3 studies (19, 21, 24) including 17 stroke patients showed a significant increase in FMA score [MD = 5.6, 95% CI (3.77, 7.5), *P* < 0.001], and the data were found to be homogenous (I^2^ = 0, *P* = 0.657) (Supplementary Figure S3).

For BCI-controlled FES, the meta-analysis of 6 studies (33–36, 38, 44) with a total of 99 patients exhibited an improvement in FMA score [MD = 5.37, 95% CI (4.2, 6.6), *P* < 0.001], along with the homogeneity in data (I^2^ = 0, *P* = 0.198) (Supplementary Figure S3).

Finally, after analyzing the data from 3 EMG-controlled FES studies (51, 56, 58) involving 60 patients, it was found that EMG-FES rehabilitation led to a significant increase in FMA score [MD = 14.14, 95% CI (11.72, 16.6), P < 0.001], and the data were homogenous (I^2^ = 0, *P* = 0.006) (Supplementary Figure S3).

#### 3.3.2 Change in action research arm test (ARAT) score

The meta-analysis of 3 EMG-based FES studies (51, 55, 56) including 49 patients indicated a statistically significant increase in the ARAT score [MD = 11.9, 95% CI (8.8, 14.9), P < 0.001], and the data demonstrated homogeneity (I^2^ = 0, *P* = 0.534) (Supplementary Figure S4).

## 4 Discussion

FES-based stroke rehabilitation systems have been increasingly used as a therapeutic tool to restore physical movements with post-stroke motor impairment. The rehabilitation outcomes may vary depending on the type of FES administered (open-loop or closed-loop). In either case (open-loop/closed-loop), the patient is instructed to actively attempt the required task, hence ensuring the cortical involvement during the training that plays a vital role in motor recovery. This review paper provides an in-depth literature review of open-loop and closed-loop FES systems for upper limb rehabilitation in terms of their design, advantages, and clinical stroke application (including RCTs and feasibility studies). We conducted a meta-analysis of the included studies to assess the effectiveness of different FES-based upper limb rehabilitation systems (Pre-defined FES, BCI-FES, and EMG-FES). Firstly, we performed the quality assessment of the included articles to ensure the high quality of the provided information. As a result, it was found that most of the articles come under the “Good” quality category. Additionally, all the studies in our analysis exhibited homogeneous data. Data homogeneity in meta-analysis suggests that the findings from individual studies are consistent with each other, thereby enhancing the reliability of drawing conclusions from the aggregated data. Moreover, the statistical analysis was performed individually on each study within sub-groups of “Pre-defined FES, BCI-controlled FES, and EMG-controlled FES”. The meta-analysis results showed that each FES-based rehabilitation system significantly improved upper limb motor function in stroke patients, as measured by FMA and ARAT scores (Supplementary Figures S3, S4). Despite comprehensive search strategies, there is a possibility of having the following limitations in our review process:

- **Incomplete Retrieval of Studies**: It is possible to miss relevant studies, especially if they are published in non-indexed journals, not available in electronic databases, or written in languages not included in the search criteria.
- **Reporting Bias**: In some cases, relevant data is incomplete or unavailable. For instance, some studies have not reported the outcome measures that are used to assess the effectiveness of interventions and track the progress of individuals recovering from a stroke. Hence, incomplete reporting of outcomes can lead to reporting bias, affecting the completeness and accuracy of the data available for analysis.

To conclude the discussion on FES-based upper limb stroke rehabilitation systems, it is important to address some key questions related to the current level of implementation, design feasibility, practical credibility, clinical considerations, and future interpretation. These questions will help clarify the current state of these systems and inform their future development.

### 4.1 Are FES based therapies more effective than non-FES conventional therapies for stroke rehabilitation?

Several studies have compared FES and non-FES upper limb stroke rehabilitation (17, 34, 35, 51, 56). In (17), a study of 30 stroke survivors (experimental FES and non-FES control group) demonstrated improvement in clinical scores, suggesting that FES reduces wrist flexor spasticity as compared to non-FES. Kim et al. (34) and Miao et al. (35) in an RCT investigated the influence of the BCI-FES system on the motor recovery of upper extremities in stroke survivors. The measured outcomes validated enhanced recovery via BCI-based system as compared to physical training. Similarly, Shindo et al. (51) and Jonsdottir et al. (56) in an RCT tested the performance of a EMG-controlled FES against non-FES conventional therapies. Following the completion of the rehabilitation sessions, the pre- and post-performance of participants were evaluated using various clinical scores such as FMA and ARAT. EMG-FES induced a greater level of improvement in comparison to the non-FES control group. In addition to EMG-controlled FES, EMG-triggered FES also shows promising results when compared with non-FES rehabilitation therapies (85–88).

### 4.2 What are the main clinical considerations for the use of electrical stimulation?

To ensure the safe use of FES in clinical applications, it is important to consider some key precautions and factors that may affect its delivery beyond the targeted muscle, leading to unexpected consequences. In (89), Marquez-Chin et al. give a complete list of clinical considerations that include:

- **Pregnancy:** The effect of FES on pregnancy or the fetus is not known and therefore, should be avoided to use (90).
- **Lesions:** The application of FES should be avoided on open skin lesions, as it can increase irritation and further damage the existing lesion (90).
- **Cardiac pacemakers:** Electrical stimulation may interfere with the electrical signals from pacemakers, potentially affecting their functioning (91).
- **Congestive heart failure conditions:** The cardiovascular demand resulting from the muscle contractions produced by the FES may require special attention before and during the delivery of stimulation (92, 93).

### 4.3 Based on the reported studies, which FES neurorehabilitation system can be considered the best among all?

There is no so-called “BEST” system, as every FES system has pros and cons, and its selection depends on the required stroke application. For instance, open-loop FES and BCI-FES can be used by stroke survivors with no muscle activity, whereas EMG-FES can only be used by the ones having residual muscle activity. However, regardless of their encouraging results, the reported FES-based rehabilitation studies contain certain limitations and shortcomings.

#### 4.3.1 No RCT is conducted

Numerous studies did not conduct randomized controlled trials; instead, they just conducted feasibility studies within the stroke population (18–24, 37–45, 58). Such studies included no control group and only performed the rehabilitation protocols on the experimental group. A control group provides a baseline against which the treatment group can be compared. Without a control group, assessing whether any observed changes are greater or different from what would naturally occur without the intervention is challenging. Also, it may be challenging to generalize the study's findings to a broader population or to other settings because there is no comparison to determine whether the effects are consistent across different contexts.

#### 4.3.2 Small sample size

Reported RCTs (17, 32–36, 51, 55, 56) and feasibility studies (19, 21, 23, 24, 38, 42, 44, 58) claimed statistical significance results; however, their sample size is not large enough (lies between 1 and 51 stroke patients in one group). According to Kaptein (94), a conventional RCT requires a group size of at least 64 individuals in each group to obtain statistically significant results. Hence, a small sample size questions the credibility and reliability of studies. It indicates that further investigation or a larger sample size may be needed to establish a more definitive relationship.

#### 4.3.3 Lack of follow-up data

Also, there was no mention of the follow-up data to determine whether the improvement was retained or not (17–24, 32–45, 58). The absence of follow-up data in rehabilitation can impede the assessment of long-term outcomes, the identification of relapses, and the ability to make informed decisions about treatment effectiveness and planning. It is crucial for both clinical practice and research to include follow-up assessments to ensure that the benefits of rehabilitation are sustained and optimized over time.

#### 4.3.4 Lack of neuroplasticity validation

When an individual experiences functional improvement, such as regaining motor skills after a stroke rehabilitation, the brain can reorganize its neural circuits and establish new connections or strengthen existing ones to support improved function (95, 96). These neuronal changes can be determined by different techniques, which mainly include electroencephalography (EEG)/evoked potentials (ERPs), structural and functional magnetic resonance imaging (MRI), and transcranial magnetic stimulation (TMS) (97). Studies (17, 19, 21, 23, 24, 32–36, 38, 42, 44, 51, 55, 56) have shown that the different FES-based rehabilitation causes functional improvement among stroke patients, but none of them has validated their findings by presenting the neuroplasticity outcomes. Thus, it remains uncertain to what extent neuroplasticity has occurred due to open/closed-loop FES rehabilitation.

Hence, it is hard to conclude which specific FES system is best. However, many research studies showed that closed-loop FES is more effective than open-loop FES for motor recovery (32–35, 38, 55). Among closed-loop FES, which system is more efficient (either BCI-FES or EMG-FES) remains unknown, as currently, no RCT has been conducted to directly compare their efficacy in neurorehabilitation. Furthermore, from the clinical implementation point of view, an open-loop FES has been widely used clinically for many years (for stroke rehabilitation), whereas closed-loop FES is mainly applied in the laboratory as a research protocol (especially BCI-FES). As per our knowledge, “RecoveriX from g.tec” (39) is the only commercially available BCI-FES system for stroke rehabilitation, which is also in its initial phases to be adopted by clinicians/therapists.

### 4.4 Can the effectiveness of FES systems be further enhanced by combining them with other systems/paradigm?

To enhance the performance of FES rehabilitation, it can either be combined with other rehabilitation systems (like robotic systems and exoskeletons) or any additional paradigm (like virtual reality), hence, developing a “Hybrid FES Rehabilitation System”.

#### 4.4.1 Hybrid with other rehabilitation systems (robotics system and exoskeleton)

In (98), the integration of electrical stimulation with robotic arm training resulted in significant improvements in the range of motion for shoulder and elbow movements in subacute stroke survivors, compared to conventional robotic training. Meadmore et al. (99) developed a new rehabilitative system, featuring FES, robotic support, and voluntary effort. The results demonstrated improvements in arm impairment among five stroke survivors. Another study (100) tested an EMG-driven FES-robotic system on 11 chronic stroke survivors to rehabilitate finger, wrist, and elbow movement. Significant improvement in physical functions and arm impairment has been obtained. Qian et al. (101) used the same FES-robotic system on 24 sub-acute stroke survivors, which showed higher motor outcomes at the distal joints than the control group (conventional therapy). Although there are potential advantages in using hybrid FES robotic systems for upper limb rehabilitation, a review study has revealed that only a limited number of hybrid systems have undergone testing with stroke survivors (102). This could be due to challenges associated with integrating both rehabilitation technologies or the absence of integrated platforms that could be user-friendly and easy to set up.

Ambrosini et al. (103) developed a novel hybrid neurorehabilitation system that integrated a passive exoskeleton (named RETRAINER) with an EMG-triggered FES unit. In (103), they tested the feasibility and functionality of the hybrid system in a clinical environment. Later, they performed a pilot study (104) and RCT (105) to test the performance of the developed system for upper limb recovery. The pilot study was implemented on seven post-acute stroke survivors. Preliminary results confirmed that the hybrid FES exoskeleton system can be used for stroke rehabilitation, positively impacting arm functional recovery (104). In (105), an RCT involving 72 stroke survivors validated the performance of a hybrid system compared to advanced conventional therapy (ACT) for task-oriented arm training. The findings showed that the hybrid FES exoskeleton system achieved a significantly better improvement in upper limb functionality.

#### 4.4.2 Hybrid with additional paradigm (virtual reality)

During the FES-based rehabilitation therapy, the participants started losing interest, and it became difficult for them to maintain the training motivation. This decline in the level of engagement could be attributed to the extended duration of the sessions, the repetitive exercises involved, and the clinical environment in which the rehabilitation took place (106). Therefore, physical therapists increasingly turn to virtual reality (VR) paradigms and incorporate VR into their neurorehabilitation protocols (107). By providing a virtual environment with thrilling, stimulating, and entertaining tasks, VR can keep participants more focused and motivated during rehab exercises, potentially engaging additional neural circuits to restore motor functions more effectively. Hence, the RecoveriX system combines VR with FES and commercially introduced hybrid VR-based BCI-FES stroke rehabilitation systems (39). Different studies (40–44) suggested that the RecoveriX system caused the improvement in upper extremity movements via stroke rehabilitation.

However, as VR is a newly adopted method in neurorehabilitation, initial testing has mostly been performed on small populations. Furthermore, low-quality VR may cause simulator sickness in stroke survivors, thus necessitating high-quality VR that replicates actual environments as realistically as possible. Thus, more research is needed to investigate the practical implementation and feasibility of hybrid VR-based FES systems for neurorehabilitation.

### 4.5 As flexible electronics (FE) is nowadays being integrated within the healthcare system, what is the emerging potential of FE combined with FES and other technologies for stroke rehabilitation?

Regarding the future of FES-based neurorehabilitation systems, it is highly likely that “Flexible Electronics” (FE) will be integrated into this field. FE is an innovative technology that offers a flexible hardware platform to perform signal amplification, precise sensing, and delivery of FES (64). Modern FES devices typically employ a pair of large gel electrodes, which generate multiple current paths, hence stimulating various muscles. This results in an inability to activate specific muscles selectively and can lead to muscle fatigue (108). To address this issue and enable selective stimulation, a flexible multiple-electrode array has been created, which can be conveniently applied to curved surfaces and cover several targeted areas at a single location (109–113). This array allows for individual electrode activation, providing selective stimulation to targeted muscles. Moreover, research has demonstrated that distributing the stimulation spatially across multiple electrodes can also delay the occurrence of muscle fatigue (114–116).

De Marchis et al. (109) used an FE array comprising 27 electrodes to administer FES. Eight healthy participants were tested using the system to execute various wrist and finger movements of the left arm. The findings indicate that the electrode array can deliver precise stimulation to specific muscles, making it a viable option for stroke rehabilitation. In (110), a flexible 24-electrode array named “e-sleeve” was built for an FES rehabilitation device. The performance of the e-sleeve was evaluated on eight stroke patients with upper limb disability for executing “hand opening and pointing” actions. Similarly, Yang et al. (111) and Loitz et al. (112) developed screen-printed fabric electrode arrays (FEAs) for a wearable FES device. The findings indicate that the FEAs can successfully facilitate desired movements, such as “open hand,” “pinch,” and “pointing” gestures. Another flexible FES electrode array was designed by Maleševic et al. (113), called “Intelligent FES (INTFES)”. It was tested on three stroke survivors to produce grasping movements. The outcomes demonstrated that INTFES activates the appropriate electrode configuration (thus, muscles) and successfully achieves grasping movements while maintaining wrist stabilization.

EEG and EMG acquisition systems are also a key part of FES rehabilitation systems, underscoring the need for flexible EEG/EMG electrodes to support advanced solutions for acquiring brain and muscle signals. FE electrodes are recommended over conventional electrodes because they can be placed on curved body surfaces and also incorporated into wearable devices of various shapes. FE electrodes enable the design of portable systems and optimize the overall compactness (117). This makes them feasible for everyday use and enables patients to carry out long-lasting rehabilitation therapies with greater ease and comfort (118). Several studies have developed and tested flexible EEG (119, 120) and EMG (121–123) electrodes for signal acquisition. Moreover, in 2019, research was published in “Nature Machine Intelligence,” in which Mahmood et al. designed a fully portable, flexible, and wireless BCI system for EEG data acquisition (124).

Thus, it is clear that FE technology is rapidly growing in healthcare; however, its neurorehabilitation application is still in its infancy as very little testing is performed on stroke survivors (mainly done on healthy subjects). In the future, there is a high chance that flexible technology will become mature enough to be largely used for designing flexible and portable stroke rehabilitation systems.

## 5 Conclusion

This systematic review provides a comprehensive overview of three types of FES systems used for post-stroke upper limb rehabilitation: Manual FES, BCI-FES, and EMG-FES. A meta-analysis has been performed that validated the effectiveness of FES-based systems for upper limb stroke rehabilitation. Among the feasibility tests and RCTs for stroke application, it provides a comprehensive understanding of the design, effectiveness, and limitations. The article also discusses some of the hybrid approaches, including robotics systems and virtual reality, which can contribute to enhancing the efficacy of FES-based rehabilitation systems. Thus, this review article will help researchers to: (1) identify the new research gaps in stroke rehabilitation; (2) assess the possibility of integrating flexible electronics and hybrid approaches while developing new FES systems in the future; (3) consider the shortcomings of previous clinical studies while designing the new rehabilitation protocols; (4) determine the usefulness of different types of FES rehabilitation approaches and perform different RCTs to compare their performance.

## Data availability statement

The original contributions presented in the study are included in the article/Supplementary material, further inquiries can be directed to the corresponding author.

## Author contributions

MK: Conceptualization, Funding acquisition, Methodology, Writing – original draft, Writing – review & editing, Visualization. HF: Writing – original draft. HG: Writing – original draft. IB: Writing – review & editing. SP: Supervision, Writing – review & editing. BR: Supervision, Writing – review & editing. ML: Supervision, Writing – review & editing. AP: Supervision, Writing – review & editing. KM: Supervision, Writing – review & editing.
