# Supplementary material for: A systematic review on functional electrical stimulation based rehabilitation systems for upper limb post-stroke recovery
Source: Front Neurol. 2023 Dec 8;14:1272992. doi: 10.3389/fneur.2023.1272992 (PMC10739305; doi:10.3389/fneur.2023.1272992)
Supplement: Supplementary file 1 [file Data_Sheet_1.PDF]

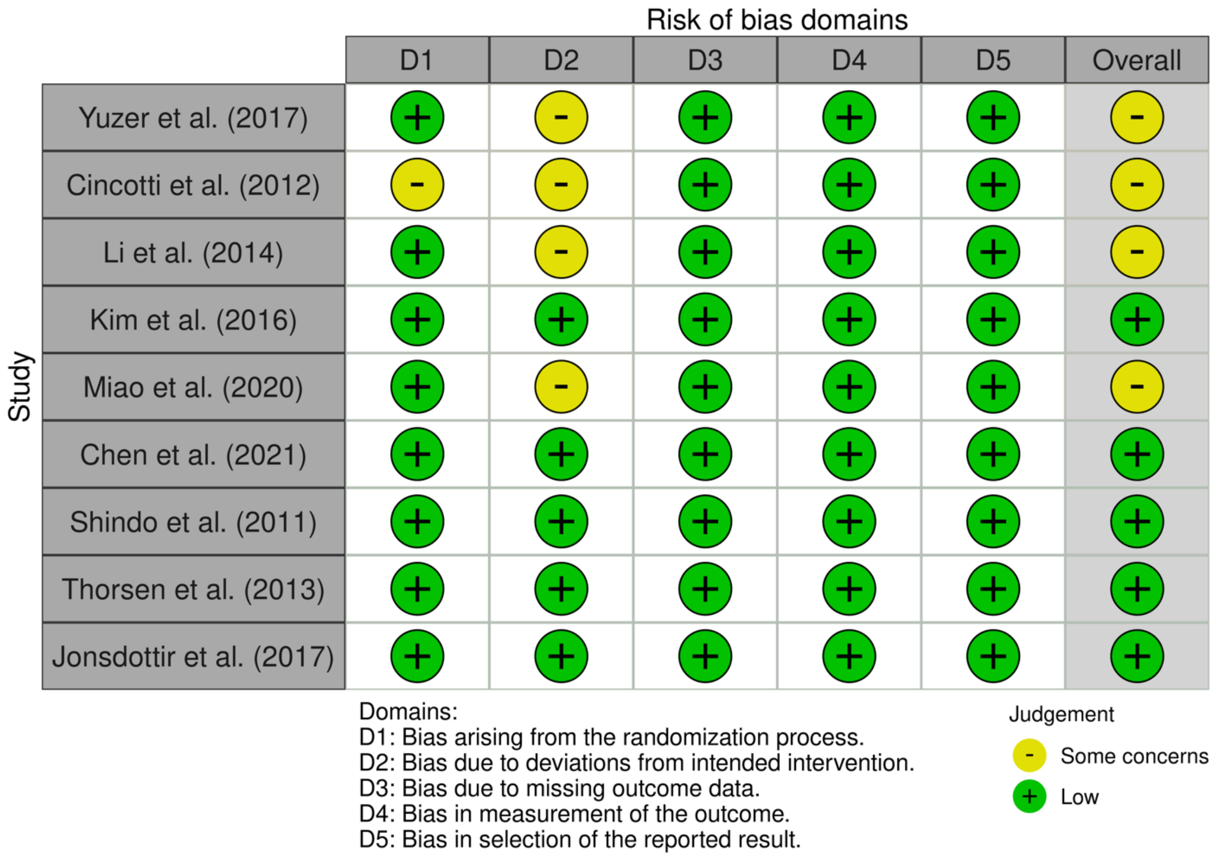

Figure S1: ROB summary of included RCTs

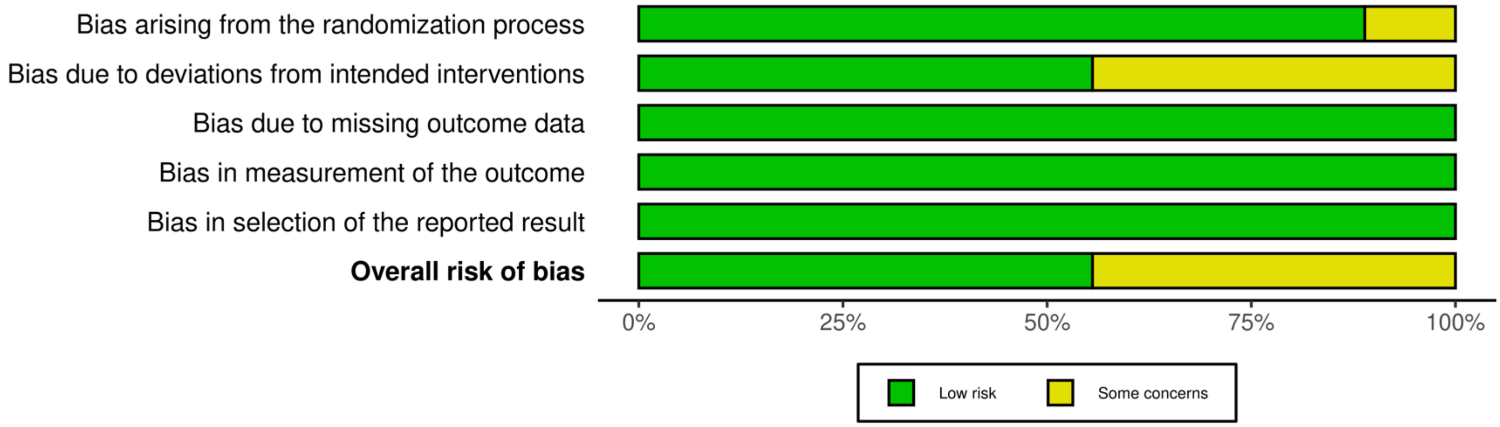

Figure S2: ROB graph of included RCTs

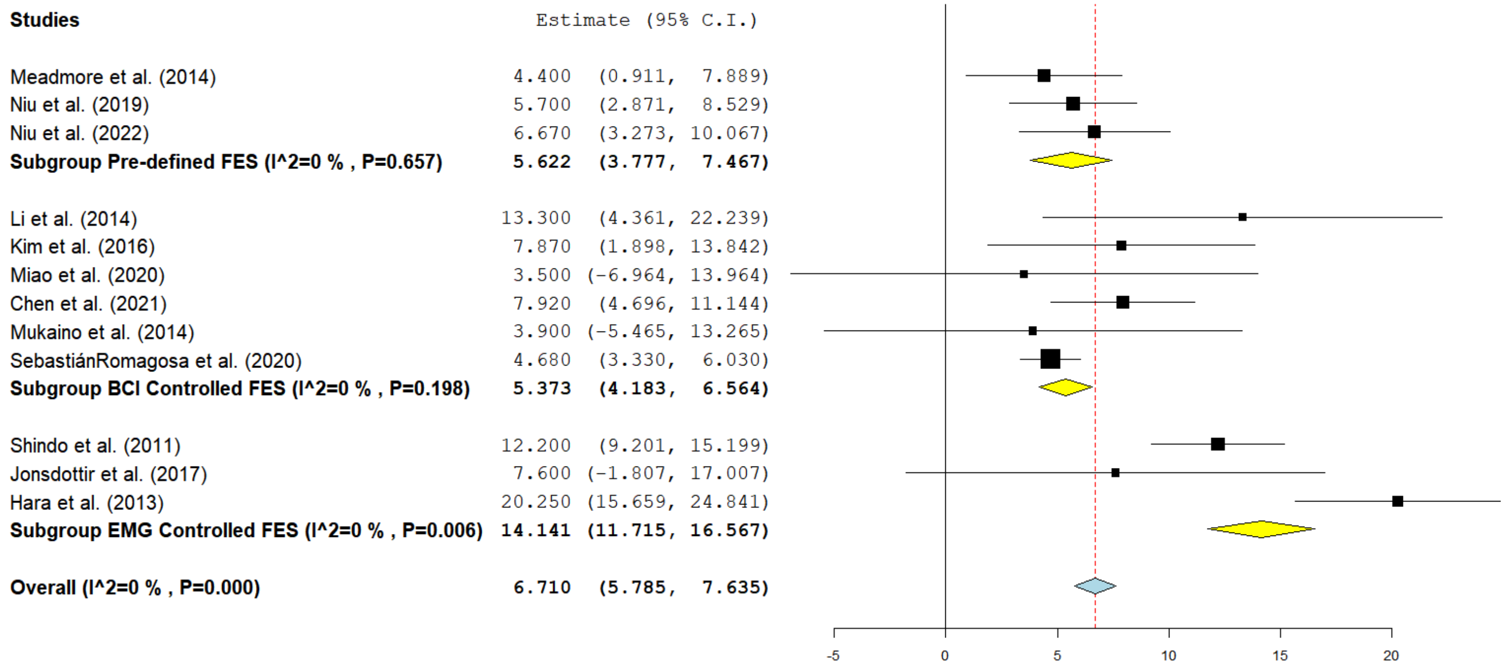

Figure S3: Forest plot of the change in FMA score

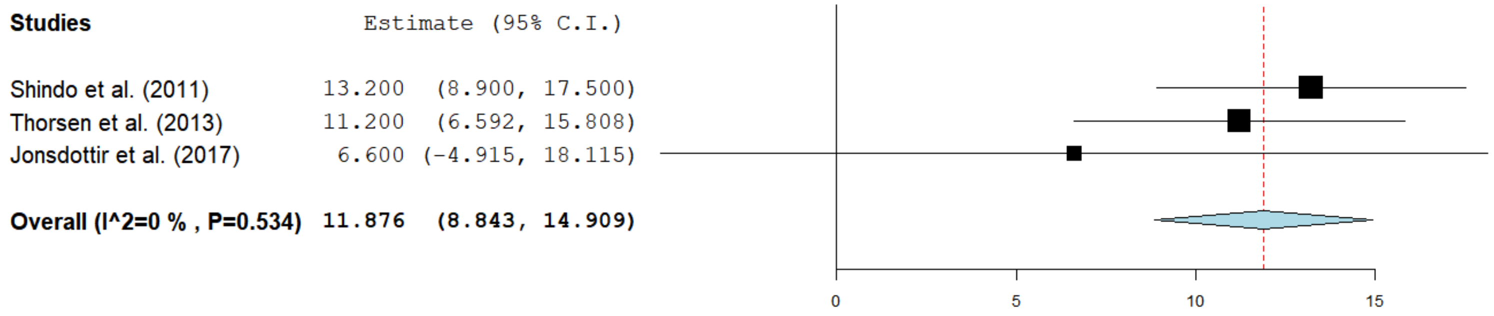

Figure S4: Forest plot of the change in ARAT score
