# Supplementary material for: A systematic review on functional electrical stimulation based rehabilitation systems for upper limb post-stroke recovery
Source: Front Neurol. 2023 Dec 8;14:1272992. doi: 10.3389/fneur.2023.1272992 (PMC10739305; doi:10.3389/fneur.2023.1272992)
Supplement: Supplementary file 2 [file Data_Sheet_2.PDF]

Table ST1: Quality assessment of case series studies using NIH tool.

Study ID	NIH Quality Assessment Tool for Observational Case Series Studies									Overall quality
	9. Was the study question or objective clearly stated?	2. Was the study population clearly and fully described, including a case definition?	3. Were the cases consecutive?	4. Were the subjects comparable?	5. Was the intervention clearly described?	6. Were the outcome measures clearly defined, valid, reliable, and implemented consistently across all study participants?	7. Was the length of follow-up adequate?	8. Were the statistical methods well-described?	9. Were the results well-described?	
**Makowski et al. (2014) [18]**	Yes	Yes	Yes	No	Yes	Yes	Yes	No	Yes	Fair
**Meadmore et al. (2014) [19]**	Yes	Yes	No	Yes	Yes	Yes	Yes	Yes	Yes	Good
**Sun et al. (2018) [20]**	Yes	Yes	No	No	Yes	Yes	Yes	No	Yes	Fair
**Niu et al. (2019) [21]**	Yes	Yes	Yes	No	Yes	Yes	Yes	No	Yes	Fair
**Chou et al. (2020) [22]**	Yes	Yes	Yes	Yes	Yes	Yes	Yes	No	Yes	Good
**Niu et al. (2022) [24]**	Yes	Yes	Yes	Yes	Yes	Yes	Yes	No	Yes	Good
**Irimia et al. (2017) [41]**	Yes	Yes	Yes	No	Yes	Yes	Yes	No	Yes	Fair
**Qiu et al. (2018) [43]**	Yes	Yes	Yes	No	Yes	Yes	Yes	Yes	Yes	Good
**SebastiánRomagosa et al. (2020) [44]**	Yes	Yes	Yes	No	Yes	Yes	Yes	Yes	Yes	Good
**Choi et al. (2020) [45]**	Yes	Yes	Yes	No	Yes	Yes	Yes	Yes	Yes	Good
**Hara et al. (2013) [58]**	Yes	Yes	Yes	No	Yes	Yes	Yes	Yes	Yes	Good

Table ST2: Quality assessment of case reports using the Joanna Briggs Institute (JBI) Critical Appraisal tool

Study ID	1. Were patient's demographic characteristics clearly described?	2. Was the patient's history clearly described and presented as a timeline?	3. Was the current clinical condition of the patient on presentation clearly described?	4. Were diagnostic tests or assessment methods and the results clearly described?	5. Was the intervention(s) or treatment procedure(s) clearly described?	6. Was the post-intervention clinical condition clearly described?	7. Were adverse events (harms) or unanticipated events identified and described?	8. Does the case report provide takeaway lessons?	Overall quality
**Martín Odriozola et al. (2021) [23]**	Yes	Yes	Yes	Yes	Yes	Yes	Yes	Yes	Good
**Daly et al. (2009) [37]**	Yes	Yes	Yes	Yes	Yes	Yes	Yes	Yes	Good
**Mukaino et al. (2014) [38]**	Yes	Yes	Yes	Yes	Yes	Yes	Yes	Yes	Good
**Sabathiel et al. (2016) [40]**	Yes	Yes	Yes	Yes	Yes	Yes	Yes	Yes	Good
**Cho et al. (2017) [42]**	Yes	Yes	Yes	Yes	No	Yes	Yes	Yes	Good
